# Blockade of SDF-1/CXCR4 signalling inhibits pancreatic cancer progression *in vitro* via inactivation of canonical Wnt pathway

**DOI:** 10.1038/sj.bjc.6604745

**Published:** 2008-10-28

**Authors:** Z Wang, Q Ma, Q Liu, H Yu, L Zhao, S Shen, J Yao

**Affiliations:** 1 Department of Hepatobiliary Surgery, The First Affiliated Hospital of Xi'an Jiaotong University, Xi'an 710061, China

**Keywords:** CXCR4, Wnt, pancreatic cancer progression, RNAi

## Abstract

Extra-pancreatic metastasis is a difficult problem for surgical intervention in pancreatic cancer. CXC chemokine receptor 4 (CXCR4) was considered to have an important role in this process. We hypothesized it may contribute to the pancreatic cancer progression through influencing canonical Wnt pathway. The purpose of this study was to examine the functional role of CXCR4 in the progression of pancreatic cancers and explore the possible mechanism. To this end, the relation between CXCR4 and clinical characteristics was analysed. shRNA against CXCR4 was applied to disrupt the SDF-1/CXCR4 signal transduction pathways in pancreatic cancer cell lines. Our results showed that overall survival in the case of patients positive for CXCR4 expression was significantly lower than that in the case of patients negative for CXCR4 expression. Notably, *in vitro* studies we observed that the abrogation of CXCR4 could obviously influence the pancreatic cancer cell phenotype including cell proliferation, colony formation, cell invasion and also inhibit the TOPflash activity. In addition, Wnt target genes and mesenchymal markers such as Vimentin and Slug were also inhibited in CXCR4 knockdown cells. Collectively, these data reported here demonstrate CXCR4 could modulate the canonical Wnt pathway and perhaps be a promising therapeutic target for pancreatic cancer progression.

Pancreatic ductal adenocarcinoma (PDA) is the fourth leading cause of cancer deaths in Western countries (Jemal *et al*, 2007). It represents a disease with a poor prognosis, and the survival has been further associated with tumours that locally extended beyond the pancreas and metastasize in the regional lymph nodes (Butturini *et al*, 2008). The reasons behind the peculiar metastatic tropism are largely unknown. It appears that some understanding of the possible mechanism underlying the progression of pancreatic cancer is required to improve the clinical outcome.

Metastasis progress is a complex mechanism in which many factors can potentially influence tumour dissemination (Murphy, 2001). Recently, the function of chemokines in the process of cancer metastasis has attracted increased attention. Chemokines and their receptors are critical mediators of cell migration during routine immune surveillance, inflammation, and development (Singh *et al*, 2007; O'Hayre *et al*, 2008). One of the best studied chemokine receptors is CXCR4, primarily because of its role as a coreceptor for HIV entry as well as its ability to mediate the metastasis of a variety of cancers (Balkwill, 2004a). CXCR4 selectively binds the CXC chemokine stromal cell-derived factor 1 (SDF-1, or CXCL12), which has been found to play an important role in tumorigenesis, proliferation, metastasis, and angiogenesis in cancers (Burger and Kipps, 2006). The expression of CXCR4, confirmed in more than 23 solid cancers, was upregulated on the surface of tumour cells of epithelial origin (Balkwill, 2004b). Moreover, CXCR4-positive tumour cells could migrate toward distant organs in response to SDF-1 gradient. With the inhibition of CXCR4, the growth and invasion could be impaired in some type of cancer cells (Muller *et al*, 2001; Ottaiano *et al*, 2005). Involvement of SDF-1/CXCR4 was reported during prostate cancer, ovarian cancer, colorectal cancer, hepatocellular carcinoma as well as pancreatic cancer (Hart *et al*, 2005; Jiang *et al*, 2006; Schimanski *et al*, 2006; Yoshitake *et al*, 2008). It was reported that CXCR4 was expressed at higher levels in pancreatic cancer cells and could influence the clinical outcome of PDA patients (Wehler *et al*, 2006; Billadeau *et al*, 2006). The microarray results showed that pancreatic juice from pancreatic cancer patients contained increased RNA level of the CXCR4 gene (Rogers *et al*, 2006). Recent evidence has shown that multiple factors could enhance CXCR4 expression, such as hypoxia-inducible factor 1, vascular endothelial growth factor, activation of nuclear factor kappa B and hepatocyte growth factor (Bachelder *et al*, 2002; Staller *et al*, 2003; Helbig *et al*, 2003; Matteucci *et al*, 2005). However, the relative mechanism underlying the downstream modulation function of SDF-1/CXCR4 on pancreatic cancer progression is poorly understood.

The Wnt/*β*-catenin canonical Wnt-signalling pathway has been implicated in tumorigenesis at several sites, including the colon, rectum, breast, and liver (Peifer and Polakis, 2000). Its central component, namely, *β*-catenin, plays a critical role in this process (Nelson and Nusse, 2004). Firstly, *β*-catenin is phosphorylated in the inactive phase of Wnt pathway with the absence of Wnt or the presence of Wnt inhibitors. The aberrant secretion of Wnt factors or the presence of APC mutation could lead to abnormal *β*-catenin activation which in turn leads to the activation of Wnt signalling and the process of epithelial–mesenchymal transition (EMT) (Brabletz *et al*, 2001; Reya and Clevers, 2005). A number of downstream target genes of Wnt/*β*-catenin signalling have been reported, which play critical roles in carcinogenesis by affecting cell growth, cell cycling, cell survival and invasion (www.stanford.edu/~rnusse/wntwindow.html). The mutation of APC or *β*-catenin which is commonly found in other gastrointestinal cancer could not be always detected in PDA (Abraham *et al*, 2002). Aberrant activation of Wnt signalling is a significant feature of human pancreatic adenocarcinoma, as revealed by aberrant *β*-catenin expression in a significant fraction (30–65%) of tumours (Lowy *et al*, 2003; Al-Aynati *et al*, 2004; Zeng *et al*, 2006).

Based on previous research on the central role of CXCR4 and the canonical Wnt pathway, we hypothesized that CXCR4 plays a role in the pancreatic cancer metastasis through the Wnt/*β*-catenin pathway (Wang and Ma, 2007). The aim of this study was to determine the role of CXCR4 in pancreatic cancers and elucidate the underlying mechanism.

## Materials and methods

### Cell line and culture conditions

Human pancreatic cancer cell lines (PC-2, PC-3, Miapaca-2, Bxpc-3, and Panc-1) were stored in the Department of Hepatobiliary Surgery, the First Affiliated Hospital of Xi'an Jiaotong University. The cells were cultured in DMEM (Invitrogen, Carlsbad, CA, USA) supplemented with 10% FBS (Invitrogen, Carlsbad, CA, USA), penicillin (100 U ml^−1^) and streptomycin (0.1 mg ml^−1^).

### Immunohistochemistry

Samples including 48 pancreatic carcinoma and 8 normal pancreas specimen were obtained from the Department of Hepatobiliary Surgery, the First Affiliated Hospital of Xi'an Jiaotong University. Immunohistochemical staining for CXCR4 were performed using the SABC kit (Maxim, Fuzhou, China) according to the manufacturer's instruction. Primary antibody for CXCR4 (1 : 50) was obtained from eBioscience (San Diego, CA, USA) and incubation overnight at 4°C. For the evaluation of protein expression, the staining intensity was graded as 0 (negative), 1 (weak), 2 (medium), or 3 (strong). The extent of staining was graded as 0 (0%), 1 (1–10%), 2 (11–50%), 3 (51–80%) and 4 (>81%) according to the percentage of positive staining area relative to the total tumour area. The final immunohistochemical staining score was obtained by multiplying the intensity and the extent of staining (Leo *et al*, 2006).

### CXCR4 shRNA expression vector construct

The human CXCR4 gene sequence (Genbank accession, no. NM_003467) was analysed for a potential siRNA target with the web-based siRNA target finder and design tool provided by Genscript (Piscataway, NJ, USA) and Ambion (Applied Biosystems, Austin, TX, USA) according to the manufacturer's protocol. The following shRNA insert sequence was synthesized: 5′-gatccTGAGAAGCATGACGGACAAttcaagagaTTGTCCGTCAT GCTTCTCAttttttggaaa-3′ (target position: 301) and 5′-gatccAGCGAGGTGGACATTCATCttcaagagaGATGAATGTCCACC TCGCTttttttggaaa-3′ (target position: 1093). As a negative control, a vector was also designed, whose inserted sequence was 5′-gatccTTCTCCGAACGTGTCACGTttcaagagaACGTGACA CGTTCGGAGAAttttttggaaa-3′, which does not target any region in human genome. Sense and antisense oligonucleotides were annealed. After digestion with *Bam*HI and *Hin*dIII, the fragments were inserted into the shRNA expression vector pRNAT-U6.1/Neo (GenScript Corp., Piscataway, NJ, USA) and confirmed by plasmid sequencing.

### Stable transfection of CXCR4 shRNA expression vector

In a 24-well plate, 5 × 10^4^ cells were plated per well 1 day before transfection. Transfection was performed using the lipofectamine 2000 (Invitrogen, Carlsbad, CA, USA) according to the manufacturer's suggestion. At 24 h after transfection, the cell solution was diluted at 1 : 10 and regenerated. In the 10% DMEM culture, 400 *μ*g ml^−1^ G418 (Invitrogen, Carlsbad, CA, USA) was added for selection after the cells adhered to the plate. A limited dilution was used in a 96-well plate for repeated colony selection. After 14 days, G418 (200 *μ*g ml^−1^) was added for the future stable transfected cell culture.

### Quantitative real-time polymerase chain reaction (QT–PCR) to detect mRNA expression

To extract total RNA, 2 × 10^5^ cells were harvested with the Trizol Reagent (Invitrogen, Carlsbad, CA, USA). cDNA synthesis was conducted as followed with the SYBR® ExScript™ RT–PCR kit (Takara Biotechnology Co. Ltd., Dalian, China) according to manufacturer's instruction: 500 ng total RNA was mixed with 2 *μ*l of 5 × ExScript™ RTase buffer, 0.5 *μ*l of dNTP mixture, 0.5 *μ*l of random hexamers, 0.25 *μ*l of ExScript™ Rtase, and 0.25 *μ*l of RNase inhibitor in a total volume of 10 *μ*l. The reactions were performed at 42°C for 12 min, followed by inactivation of the reverse transcriptase at 95°C for 2 min. The cDNA was stored at −20°C. QT–PCR was performed on an ABI PRISM® 7300 Sequence Detection System (Applied Biosystems, Foster City, CA, USA) with the SYBR Green Master Mix. The final reaction volume was 25 *μ*l and contained 12.5 *μ*l 2 × SYBR® Premix Ex Taq™, 1.0 *μ*l of each primer (10 *μ*M), 0.5 *μ*l 50 × ROX reference dye, and 1.0 *μ*l cDNA. The cycling conditions were as follows: initial denaturation at 95°C for 10 min, followed by 40 cycles of 95°C for 15 s, and 60°C for 60 s. Each measurement was performed in triplicate, and no-template controls were included for each assay. In each QT–PCR, a dissociation curve analysis was conducted. GAPDH was applied as the internal housekeeping gene control. Relative gene expression was calculated using the 2^−ΔΔ*C*_t_^ method (Livak and Schmittgen, 2001).

### Cell proliferation assay

Cells were seeded in a 96-well plate at a concentration of 5 × 10^3^ per well a day before the experiment. 3-[4,5-Dimethylthiazol-2-yl]-2,5-diphenyltetrazolium bromide (MTT, 0.5 mg ml^−1^, Sigma-Aldrich, St. Louis, MO, USA) was added to each well at 1, 2, 3, 4, 5 days after seeding. Generally, cells were cultured at 37°C for 4 h, and then 150 *μ*l DMSO was added. The absorption value was measured at a wavelength of 490 nm.

### Flow cytometric analysis

Before flow cytometric analysis, 1 × 10^6^ cells were collected, washed two times with PBS, and fixed with ice-cold 70% ethanol for 24 h at 4°C. The fixed cells were stained with propidium iodide (Beckman Coulter, Miami FL, USA). After incubation for 30 m at 37°C, the samples were analysed by a Flow Cytometry. Cell cycle analysis of DNA histograms was performed with the MultiCycle software.

### Soft agar colony formation assay

In six-well plates, each well contained a bottom layer of 1% agarose, a middle layer of 0.5% agarose which included 7.5 × 10^3^ cells, and a top layer of medium. The medium at the top layer was changed on every sixth day. After 14 days, cells were stained with Giemsa solution (Gibco-BRL, Gaithersburg, MD, USA) and counted by Quantityone analysis software (BioRad Inc., Hercules, CA, USA).

### Protein extraction and western blotting

Total protein was isolated from 1 × 10^7^ cells with 200 *μ*l of ice-cold lysis buffer containing 1% Nonidet P-40 (NP-40), 50 mmol l^−1^ Tris (pH 7.4), 150 mmol l^−1^ NaCl, 0.1% sodium dodecyl sulfate (SDS), 0.5% deoxycholate, 200 *μ*g ml^−1^ phenylmethanesulfonyl fluoride (PMSF), and 50 *μ*g ml^−1^ aprotinin. Insoluble materials were removed by centrifugation at 15 000 *g* for 15 min at 4°C. The concentration of the extracted protein was measured spectrophotometrically with Coomassie G-250. Clarified protein lysates (30–80 *μ*g) were electrophoretically resolved on a denaturing SDS-polyacrylamide gel (8–12%), and electrotransferred onto nitrocellulose membranes. The membranes were initially blocked with 5% nonfat dry milk in Tris-buffered saline (TBS) for 2 h and then probed with primary antibodies against the specific protein and GAPDH (as loading control). After coincubation with the primary antibodies at 4°C overnight, the membranes were hybridised with the secondary alkaline phosphatase-conjugated goat anti-rabbit antibody (1 : 2000), goat anti-mouse antibodies (1 : 3000) (Kangchen, Shanghai, China) for 2 h at room temperature. The immunopositive bands were examined by an enhanced chemiluminescence (ECL) detection system (Amersham Bioscience, Piscataway, NJ, USA) and the images were transferred onto an X-ray film. The western blot was graded positive if the band of interest was present at the expected molecular weight corresponding to each marker protein. All analyses were done in duplicate. Antibodies used in this study: CXCR4 (1 : 200) and MMP-9 (1 : 250) (NeoMarkers, Fremont, CA, USA), Vimentin (1 : 200) and GAPDH (1 : 400) (Santa Cruz Biotechnology, Santa Cruz, CA, USA), P339-CXCR4 antibody (1 : 200) was kindly provided by Professor Joshua B. Rubin in Washington University School of Medicine at St Louis.

### *β*-Catenin/Tcf transcription reporter assay

Briefly, 1 × 10^5^ cells were seeded per well in a 24-well plate before transient transfection with the construct TOPflash or FOPflash reporter plasmid (Millipore, Billerica, MA, USA). TOPflash comprised three copies of the Tcf/Lef sites upstream of a thymidine kinase (TK) promoter and the Firefly luciferase gene. FOPflash comprised three mutated copies of Tcf/Lef sites and were used as a control for measuring nonspecific activation of the reporter. All transfections were performed using 0.8 *μ*g of TOPflash or FOPflash plasmid and 2 *μ*l lipofectamine 2000. To normalise the transfection efficiency in reporter assays, the cells were cotransfected with 0.02 *μ*g of an internal control reporter plasmid, containing Renilla reniformis luciferase driven by the TK promoter. At 24 h after TOPflash or FOPflash transfection, the luciferase assay was performed with the Dual Luciferase Assay System kit (Promega Corp., Madison, WI, USA). Relative luciferase activity was reported as the fold induction after normalization for transfection efficiency.

### Matrigel invasion assay

An invasion assay was performed with a Millicell invasion chamber (Millipore, Billerica, MA, USA). The 8 *μ*m pore inserts were coated with 15 *μ*g of Matrigel (Becton Dickinson Labware, Bedford, MA, USA). Normal culture medium with or without 100 ng ml^−1^ SDF-1*α* (R&D systems Inc., Minneapolis, MN, USA) was added at the bottom chamber to induce the cancer cell lines. Cells (5 × 10^4^) were seeded in the top chamber. The Matrigel invasion chamber was incubated for 20 h in a humidified tissue culture incubator. Non-invading cells were removed from the top of the Matrigel with a cotton-tipped swab. Invading cells on the bottom surface of filter were fixed in methanol and stained with Giemsa. Invasion ability was determined by counting the stained cells.

### Statistical analysis

All statistical analyses were performed using the SPSS13.0 software. The results were presented as means±s.d. of three replicate assays. Differences between the groups were assessed by the Student's *t*-test or analysis of variance (ANOVA). *P*<0.05 was considered to indicate statistical significance.

## Results

### Association of CXCR4 expression level with pancreatic cancers

**Figure 1:**
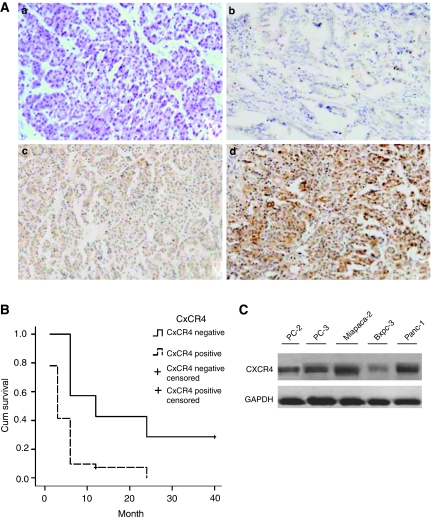
The expression of CXCR4 on pancreatic cancers. (**A**) immunohistochemistry staining of CXCR4 on normal pancreatic tissues (**a**) and pancreatic cancer tissues (**b**–**d**) × 200: negative expression (**a**), weak expression (**b**), moderate expression (**c**), strong expression (**d**); (**B**) Kaplan–Meier curve showed that the median survival time of the CXCR4-positive and CXCR4-negative groups was 5.5 and 13.5 months; (**C**). Western blot analysis revealed the CXCR4 expression for 5 pancreatic cancer cells (PC-2, PC-3, Miapaca-2, Bxpc-3, Panc-1). GAPDH was used as a loading control.

Immunohistochemistry staining results showed that CXCR4 expression was detected not in the normal pancreatic cells but in the cytoplasm of most pancreatic cancer cells. The representative staining results are shown in Figure 1A. Different CXCR4 expression was observed between the non-metastasized and metastasized cells, and observed among the tumour node metastasis (TNM) stages (*P*=0.012, 0.005, respectively) (Table 1). The median survival time of the CXCR4-positive and CXCR4-negative groups were 5.5 and 13.5 months, respectively (*P*<0.05) (Figure 1B). Moreover, CXCR4 expression was highly expressed in pancreatic cancer cell lines, especially Miapaca-2 (Figure 1C).

### Establishment of stable CXCR4 knockdown

**Figure 2:**
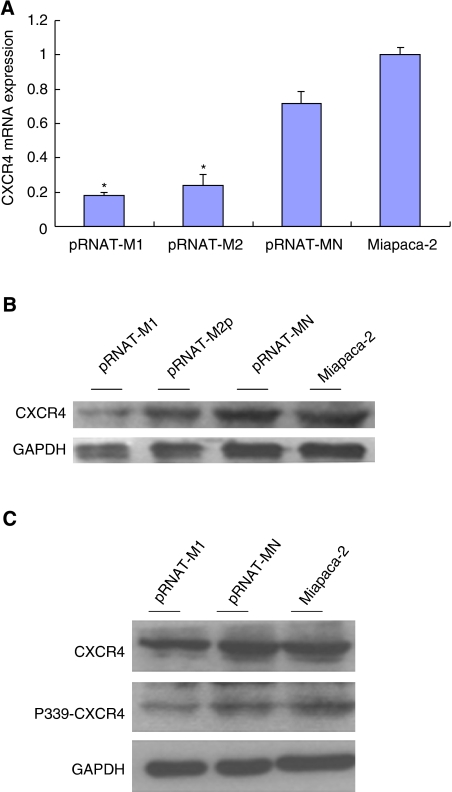
Establishment of stable CXCR4 knockdown. (**A**) QT–PCR analysis of CXCR4 expression after the transfection of different CXCR4 shRNA expression vectors: pRNAT-M1 (target position: 301), pRNAT-M2 (target position: 1093), pRNAT-MN (negative control) and Miapaca-2, ^*^P<0.05 compared with pRNAT-MN cells; (**B**) western blot with CXCR4 antibody for different transfected pancreatic cancer cells. GAPDH was detected as a loading control; (**C**) Western blot showed the different expressions of CXCR4, phosphorylated CXCR4 on Serine 339 (P339-CXCR4) on transfected cells. GAPDH was detected as a loading control.

Knockdown of transcripts using shRNA is a powerful tool for studying gene function. To study the long-term growth pattern of tumour cells *in vitro*, we developed pRNAT-U6.1/Neo vectors containing small hairpin constructs capable of generating 19 nucleotide duplex RNAi oligonucleotides. We succeeded in obtaining stable shRNA vector-transfected cells termed pRNAT-M1 (target position: 301), pRNAT-M2 (target position: 1093), and pRNAT-MN (negative control vector). QT–PCR analysis showed that compared with pRNAT-MN cells, the CXCR4 mRNA expression was inhibited up to 70% in CXCR4 shRNA-transfected cell lines, particularly in pRNAT-M1 cells (*P*<0.05). Compared with Miapaca-2 cells, approximately 20% decrease of CXCR4 mRNA expression in pRNAT-MN cells was founded (Figure 2A). Moreover, the CXCR4 protein level was also downregulated significantly in pRNAT-M1 cells than in pRNAT-MN cells (Figure 2B). Notably, the decrease of CXCR4 phosphorylation at serine 339 was observed in pRNAT-M1 cells. By band analysis, the proportion of phosphorylated CXCR4 was also reduced in pRNAT-M1 cells (Figure 2C).

### Abrogation of CXCR4 inhibits cell proliferation, delays cell cycle and decreases colony-forming ability

**Figure 3:**
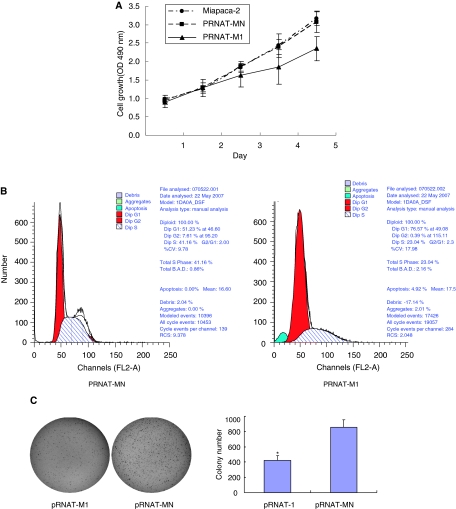
The influence of CXCR4 knockdown on the cell phenotype. (**A**) MTT assay was analysed for pRNAT-M1, pRNAT-MN, and Miapaca-2 on days 1, 2, 3, 4, and 5; (**B**) Distribution of cell cycle phases was demonstrated by flow cytometric analysis for pRNAT-M1 and pRNAT-MN; (**C**) Soft agar assay was assessed to evaluate the cell colony formation ability. The count number of the colony was shown in the diagram; ^*^P<0.05 compared with pRNAT-MN cells.

To confirm the effect of CXCR4 inhibition on cell growth, stable transfectants with CXCR4 shRNA were cultured. MTT assay showed that the pRNAT-M1 cells grew slower than pRNAT-MN cells. On the fifty day after seeding, different growth was noticed between pRNAT-M1 and pRNAT-MN cells (*P*<0.05). However, there was no difference between Miapaca-2 cells and pRNAT-MN cells (*P*>0.05) (Figure 3A). According to the analysis of cell cycle distribution by flow cytometry, we found a prolonged and prominent delay in progression from G0 to G1 phase (51.23 *vs* 76.51%), a decrease at the S phase (41.16 *vs* 23.0%) and G2-M phase (1.61 *vs* 0.39%). Moreover, sub-G1 apoptotic compartment was also observed in pRNAT-M1 cells (Figure 3B). To explore the effect of CXCR4 knockdown on tumorigenesis *in vitro*, we performed soft agar colony formation assay. It showed that the colony formation by pRNAT-M1 cells was decreased than that by pRNAT-MN cells (*P*<0.05) (Figure 3C).

### CXCR4 knockdown inhibits Wnt activity, Wnt target gene and invasion-related genes

**Figure 4:**
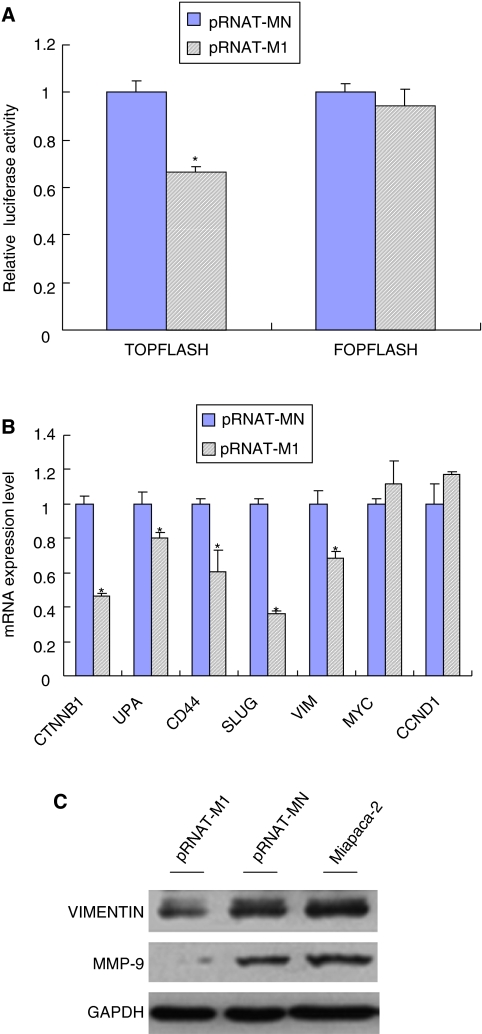
The influence of CXCR4 knockdown on Wnt target gene and invasion-related genes. (**A**) *β*-Catenin/Tcf transcription reporter assay. Normalised with control reporter plasmid, the relative luciferase activity was demonstrated. ^*^P<0.05 compared with pRNAT-MN cells; (**B**) QT–PCR analysis to examine the CTNNB1 (*β*-catenin), UPA, SLUG, CD44, MYC(c-MYC), Vim (Vimentin), CCND1 (cyclin D1) gene expression by the 2^−ΔΔ*C*_t_^ method. ^*^P<0.05 compared with pRNAT-MN cells; (**C**) western blot analysis to detect MMP-9 and Vimentin protein expression. GAPDH was used as a loading control.

As an important pathway for gastrointestinal cancer development, the Wnt/*β*-catenin pathway and genes associated with cell invasiveness were paid more attention. The *β*-catenin/Tcf transcription reporter assay was recognised as an important assessment method for evaluation of the Wnt pathway activity. As TOPflash has three TCF-binding sites, it could be applied to represent the activation of the Wnt pathway. Our data showed that compared with the pRNAT-MN cells, the CXCR4 knockdown cells exhibited a decreased TOPflash activity (*P*<0.05) and unchanged FOPflash activity (Figure 4A). Moreover, QT–PCR analysis showed that Wnt target genes such as CTNNB1, UPA, and CD44 were inhibited in pRNAT-M1 cells, but the expression of MYC and CCND1 did not change (Figure 4B). At the same time, the inhibition of CXCR4 resulted in decreased Slug, Vimentin and MMP-9 expression (*P*<0.05) (Figure 4C).

### CXCR4 knockdown decreases the invasion of pancreatic cancer cells

**Figure 5:**
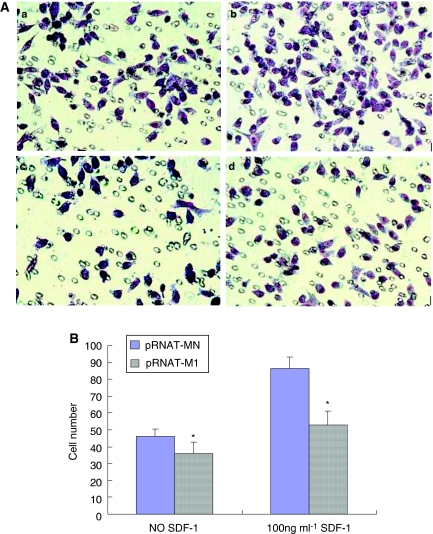
The abrogation of CXCR4 inhibits the invasive potential ability of pancreatic cancer cells. × 200; (**A**) representative staining figure: pRNAT-MN invading cells without SDF-1 stimulation (**a**), pRNAT-MN invading cells with 100 ng ml^−1^ SDF-1 stimulation (**b**), pRNAT-M1 invading cells without SDF-1 stimulation (**c**), pRNAT-M1 invading cells with 100 ng ml^−1^ SDF-1 stimulation (**d**); (**B**) the diagram of the count analysis, ^*^P<0.05 compared with pRNAT-MN cells.

The Matrigel invasion assay was performed to assess the invasiveness of the cancer cells. Representative staining results were shown at Figure 5A. The results showed that pRNAT-MN cells were more invasive than pRNAT-M1 cells (*P*<0.05). After the SDF-1 stimulation, pRNAT-MN cells showed an obvious increase in invasiveness (*P*<0.05), but pRNAT-M1 cells demonstrated a slight increase (Figure 5B).

## Discussion

The majority of PDA patients have locally advanced or metastatic disease and thus are not candidates for surgical intervention (Winter *et al*, 2006). The main cause is the extra-pancreatic invasion and metastasis to the liver, lung or other tissues. Many factors such as adhesion molecules, proteases, cytokines, and chemokine are involved in this process. A complex network of chemokine and chemokine receptors exist in tumour microenvironment. The chemokine receptor CXCR4 is one of the representative target genes (Schrader *et al*, 2002; Brand *et al*, 2005; Saur *et al*, 2005; Billadeau *et al*, 2006). In this study, we demonstrated that overall survival in the case of patients positive for CXCR4 expression was significantly lower than that in the case of patients negative for CXCR4 expression. Elevated CXCR4 expression was correlated to an advanced cancer stage and metastasis. It is similar to previous reports and also suggests that CXCR4 may be a useful marker for cancer progression (Wehler *et al*, 2006; Kajiyama *et al*, 2008).

Previous study showed siRNA directed against CXCR4 could inhibit breast cancer migration *in vitro* (Liang *et al*, 2005), but the influence of CXCR4 knockdown on pancreatic cancer was hardly known. Considering the important role of CXCR4 in cancer progression, we designed a CXCR4 shRNA vector as an alternative to blocking receptor/ligand interaction. To avoid the empty vector backbone influence, we applied the pRNAT-MN as control cells instead of Miapaca-2 cells. Our data showed that the plasmid could effectively inhibit the CXCR4 expression. At the same time, the phosphorylation of CXCR4 at serine 339 was obviously reduced, and the proportion of phosphorylated CXCR4 was decreased. Recently, it was shown that the phosphorylation of CXCR4 at serine 339 may be a way to express the CXCR4 function on the cells (Woerner *et al*, 2005), thus our data further confirmed that CXCR4 shRNA-transfected cells could effectively inhibit CXCR4 function in pancreatic cancers.

Tumorigenesis is the result of cell cycle disorganisation, leading to uncontrolled cellular proliferation and cancer progression. In this study, we demonstrated that the block of CXCR4 could not only decrease pancreatic cancer cell growth, but also increase sub-G1 apoptotic compartment, prolong the G0–G1 cycle and reduce the G2 and S phase. At the same time, it may lower anchorage-independent growth ability. With the CXCR4 knockdown, cell growth, cell cycle and cell colony formation ability were inhibited, so pancreatic cancer cell tumorigenesis was prevented effectively.

Deregulation of Wnt signalling is a well-established hallmark of certain human cancers, such as colorectal cancer and melanoma (Ilyas, 2005). Activation of the Wnt target gene will contribute to the development of cancer in terms of proliferation, invasion and metastasis, and angiogenesis (Taketo, 2006; Neth *et al*, 2007). Additionally, inhibition of Wnt signalling could reduce cell proliferation and increase apoptosis in pancreatic adenocarcinoma cells (Miao *et al*, 2003; Pasca di Magliano *et al*, 2007; Nawroth *et al*, 2007). Here, the *β*-catenin/Tcf assay showed that the TOPflash luciferase activity was obviously decreased whereas FOPflash luciferase activity has no change. Moreover, the expression of Wnt target genes including CTNNB1, CD44, UPA, and MMP-9 were markedly decreased. Thus, we assumed that the TCF-binding activity in CXCR4 knockdown cells could be effectively inhibited, which may influence the Wnt/*β*-catenin signalling and the Wnt target gene could not be activated. However, as the canonical Wnt pathway and *β*-catenin were regulated by multiple factors together, only blocking CXCR4 expression could not totally inhibit Wnt pathway.

Next, we evaluated the expression of invasion-related genes – Vimentin and Slug expression. These two genes are typical mesenchymal markers associated with the EMT process, influencing carcinoma metastasis (Thiery, 2002; Shioiri *et al*, 2006). Our data showed that Vimentin and Slug could be downregulated in CXCR4 knockdown cells. It is consistent with the study on oral carcinomas which showed that CXCR4 could influence EMT formation and cancer invasion (Onoue *et al*, 2006).

Among the chemokine and chemokine receptors interaction, SDF-1 was proved to be a stimulator of invasion for CXCR4-positive cancer cells (Kollmar *et al*, 2007). To determine whether the effect of CXCR4 shRNA on metastasis was related to pancreatic cancer *in vitro*, we conducted the Matrigel chamber invasion assay. Our data showed under normal conditions, the invasive ability of CXCR4-transfected cancer cells was inhibited compared with the control cells. Moreover, much more difference was observed between control cells and CXCR4 knockdown cells after SDF-1 stimulation. However, we also found that as there was 70% inhibition of CXCR4 through shRNA, the invasive ability could not be totally blocked.

CXCR4 was a promising marker for pancreatic cancer progression. The CXCR4-positive expression was considered to be associated with a poor clinical outcome. Moreover, the abrogation of CXCR4 could influence the pancreatic cancer cell phenotype including cell proliferation, colony formation, and cell invasion through the inhibition of canonical Wnt pathway. Given the important role that CXCR4 plays in the process of pancreatic cancer progression, CXCR4 is a very intriguing therapeutic target. Understanding the precise mechanisms of CXCR4 function should provide insight into attractive pancreatic cancer therapy.

## Figures and Tables

**Table 1:** The relation between CXCR4 expression and clinical characteristics in PDA patients

**Clinical pathologic variables**	**CXCR4 median score**	***P*-value**
*Age*		
>65	5.6	0.542
<65	4.4	
		
*Histology*		
Well	3.1	
Moderate	4.9	0.062
Poor	5.5	
		
*Lymph node metastasis*		
Positive	6.1	0.012
Negative	3.0	
		
*TNM stage*		
I	1.7	
II	3.8	0.005
III	5.4	
IV	7.9	
